# Challenges in the control of COVID-19 outbreaks caused by the delta variant during periods of low humidity: an observational study in Sydney, Australia

**DOI:** 10.1186/s40249-021-00926-0

**Published:** 2021-12-23

**Authors:** Michael P. Ward, Yuanhua Liu, Shuang Xiao, Zhijie Zhang

**Affiliations:** 1 Sydney School of Veterinary Science, The University of Sydney, Camden, NSW Australia; 2 School of Public Health, Fudan University, Shanghai, China; 3 Department of HIV/STD Prevention and Control, Shanghai Municipal Centre for Disease Control and Prevention, Shanghai, China

**Keywords:** Meteorological factor, Climate, Humidity, Temperature, SARS-CoV-2, COVID-19, Australia

## Abstract

**Background:**

Since the appearance of severe acute respiratory coronavirus 2 (SARS-CoV-2) and the coronavirus disease 2019 (COVID-19) pandemic, a growing body of evidence has suggested that weather factors, particularly temperature and humidity, influence transmission. This relationship might differ for the recently emerged B.1.617.2 (delta) variant of SARS-CoV-2. Here we use data from an outbreak in Sydney, Australia that commenced in winter and time-series analysis to investigate the association between reported cases and temperature and relative humidity.

**Methods:**

Between 16 June and 10 September 2021, the peak of the outbreak, there were 31,662 locally-acquired cases reported in five local health districts of Sydney, Australia. The associations between daily 9:00 am and 3:00 pm temperature (°C), relative humidity (%) and their difference, and a time series of reported daily cases were assessed using univariable and multivariable generalized additive models and a 14-day exponential moving average. Akaike information criterion (AIC) and the likelihood ratio statistic were used to compare different models and determine the best fitting model. A sensitivity analysis was performed by modifying the exponential moving average.

**Results:**

During the 87-day time-series, relative humidity ranged widely (< 30–98%) and temperatures were mild (approximately 11–17 °C). The best-fitting (AIC: 1,119.64) generalized additive model included 14-day exponential moving averages of 9:00 am temperature (*P* < 0.001) and 9:00 am relative humidity (*P* < 0.001), and the interaction between these two weather variables (*P* < 0.001). Humidity was negatively associated with cases no matter whether temperature was high or low. The effect of lower relative humidity on increased cases was more pronounced below relative humidity of about 70%; below this threshold, not only were the effects of humidity pronounced but also the relationship between temperature and cases of the delta variant becomes apparent.

**Conclusions:**

We suggest that the control of COVID-19 outbreaks, specifically those due to the delta variant, is particularly challenging during periods of the year with lower relative humidity and warmer temperatures. In addition to vaccination, stronger implementation of other interventions such as mask-wearing and social distancing might need to be considered during these higher risk periods.

**Graphical Abstract:**

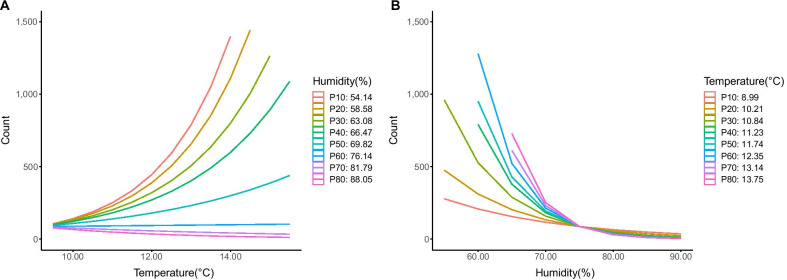

**Supplementary Information:**

The online version contains supplementary material available at 10.1186/s40249-021-00926-0.

## Background

Severe acute respiratory coronavirus 2 (SARS-CoV-2), the cause of the coronavirus disease 2019 (COVID-19) pandemic, spreads among people predominantly via respiratory droplets and aerosols, as well as fomites [1] and possibly fecal-oral [2]. Although the effect of weather on the airborne spread of SARS-CoV-2 has been investigated in some detail [3], the conclusions are inconsistent. Qi et al. [4] found that temperature had significantly negative associations with COVID-19 incidence in China using province-based data, but Guo et al. [5] showed that warmer weather might not affect COVID-19 spread based on city-scale data from China. In contrast, evidence from Jakarta, Indonesia showed that increased temperature was an environmental driver of COVID-19 outbreaks [6]; a similar conclusion was made in a global study of 190 countries conducted on data from early in the pandemic [7]. Similar contradictory results exist for humidity [6, 8–11]. One explanation is that interactions exist between temperature and humidity and their effect on SARS-CoV-2 transmission, so that the roles of these factors might be heterogenous depending on different climatic zones [12]. But whether and how the interaction among weather factors affects the spread of SARS-CoV-2 remains unclear.

Of more concern, the B.1.617.2 (delta) variant of SARS-CoV-2, first detected in India in December 2020, has spread throughout most of the world during 2021 to become the dominant strain of SARS-CoV-2 [13]. On 16 June, 2021 New South Wales Health was notified of a new COVID-19 case who was residing in Sydney’s eastern suburbs. The case, a man in his 60s, had no recent overseas travel history. However it was noted that he worked as a driver, which included transporting international flight crew [14]. Subsequently, an outbreak of COVID-19 caused by local-transmission of the delta variant commenced during early winter (June) in Sydney. To our knowledge, no studies have reported the relationship between weather and transmission of this new variant of SARS-COV-2 specifically in the southern hemisphere winter. Thus, the aim of the present study was to describe the association between reported cases of COVID-19 due to the delta variant and temperature and relative humidity.

Whilst this study builds on previous research conducted in Sydney in 2020, it also extends our knowledge about how temperature and humidity interact in terms of COVID-19 outbreaks, facilitating rational explanations for previously inconsistent study results and the behavior of a new SARS-CoV-2 variant in a predominantly susceptible population during winter, a risk period during which transmission is likely facilitated.

## Methods

### Case data

Case reports from the local health districts (LHDs) of Sydney, Australia from the beginning of the SARS-CoV-2 delta variant outbreak on 16 June to 30 September 2021 were accessed [15]. COVID-19 surveillance is based on testing (PCR on nasal and oral swabs) contacts of confirmed cases and also testing symptomatic patients. Prior to the index case, the most recent locally-acquired case had been identified on 5 May 2021. During the study period, whole genome sequencing was used to identify variants of concern on a proportion of samples from confirmed cases, and only the delta variant was found. More broadly between 29 November 2020 and 2 October 2021, a total of 11,166 cases (about 21% of all cases) were subject to whole genome sequencing and of these 11,173 (> 99.9%) were identified as the delta variant; only 6 cases of the alpha variant and one case of the beta were identified [16]. Although it is unlikely that all cases that occurred during the study period were ascertained in the surveillance system, this likely represents minor non-differential bias.

**Fig. 1:**
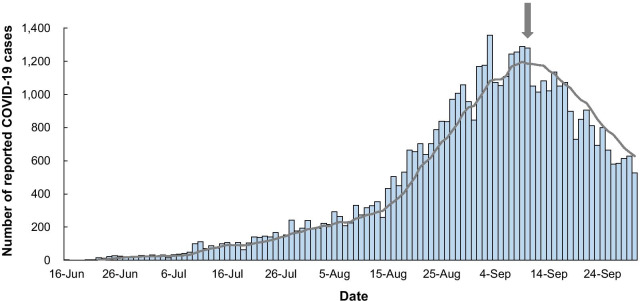
Time-series plot of cases of COVID-19 due to SARS-CoV-2 delta variant reported from Sydney, Australia during the period of 16 June and 30 September, 2021. The grey line indicates the 7-day moving average of reported cases, and the peak of the outbreak (10 September) is indicated by the arrow

Those cases whose infection source was reported to be locally-acquired, and whose LHD of residence was reported as Northern Sydney, South Eastern Sydney, South Western Sydney, Sydney or Western Sydney LHD, were included. A daily time-series of cases was created as the sum of cases reported each day. Daily reports were plotted and using a 7-day moving-average, the peak of the outbreak (1181 cases) was identified on 10 September, 2021 (Fig. 1). Therefore, further analysis was focused on the period from the beginning of the outbreak to its peak, a period of 87 days.

### Descriptive analysis

Based on the reported LHD, cases were linked to weather observation stations [17]. Daily observations of temperature (°C) and relative humidity (%) recorded at 9:00 am and at 3:00 pm during June to September were accessed [17]. Additional series of daily differences between 9:00 am and 3:00 pm temperature and 9:00 am and 3:00 pm relative humidity were created to indicate the variations. In addition, daily reported number of COVID-19 tests and number of COVID-19 vaccinations administered [18], and the mobility index for Sydney [19], were accessed and time-series created. These variables were included for descriptive purposes only, to provide context regarding this outbreak of COVID-19 caused by the SARS-CoV-2 delta variant.

### Statistical modelling

The same method used in previous studies was applied to investigate the relationship between reported cases of COVID-19 and weather variables [4, 8, 9, 12, 20]. A Spearman correlation (ρ) coefficient matrix was first calculated to avoid multicollinearity among the predictor variables, and a 14-day exponential moving average (EMA) was used to represent the effects of weather factors. Then, a univariable generalized additive model (GAM) was fitted and variables with a *P*-value < 0.1 were selected for multivariable analysis. Different multivariable analysis models were then fit to analyze the relationship between the selected weather variables and cases of COVID-19. Akaike information criterion (AIC) and likelihood ratio test were used to compare different models and determine the best model. R4.0.1 software [21] was used to perform all analyses.

A sensitivity analysis was performed by modifying the EMA (14 days to 7 or 21 days) to verify the robustness of model results.

## Results

### Epidemic characteristics

**Fig. 2:**
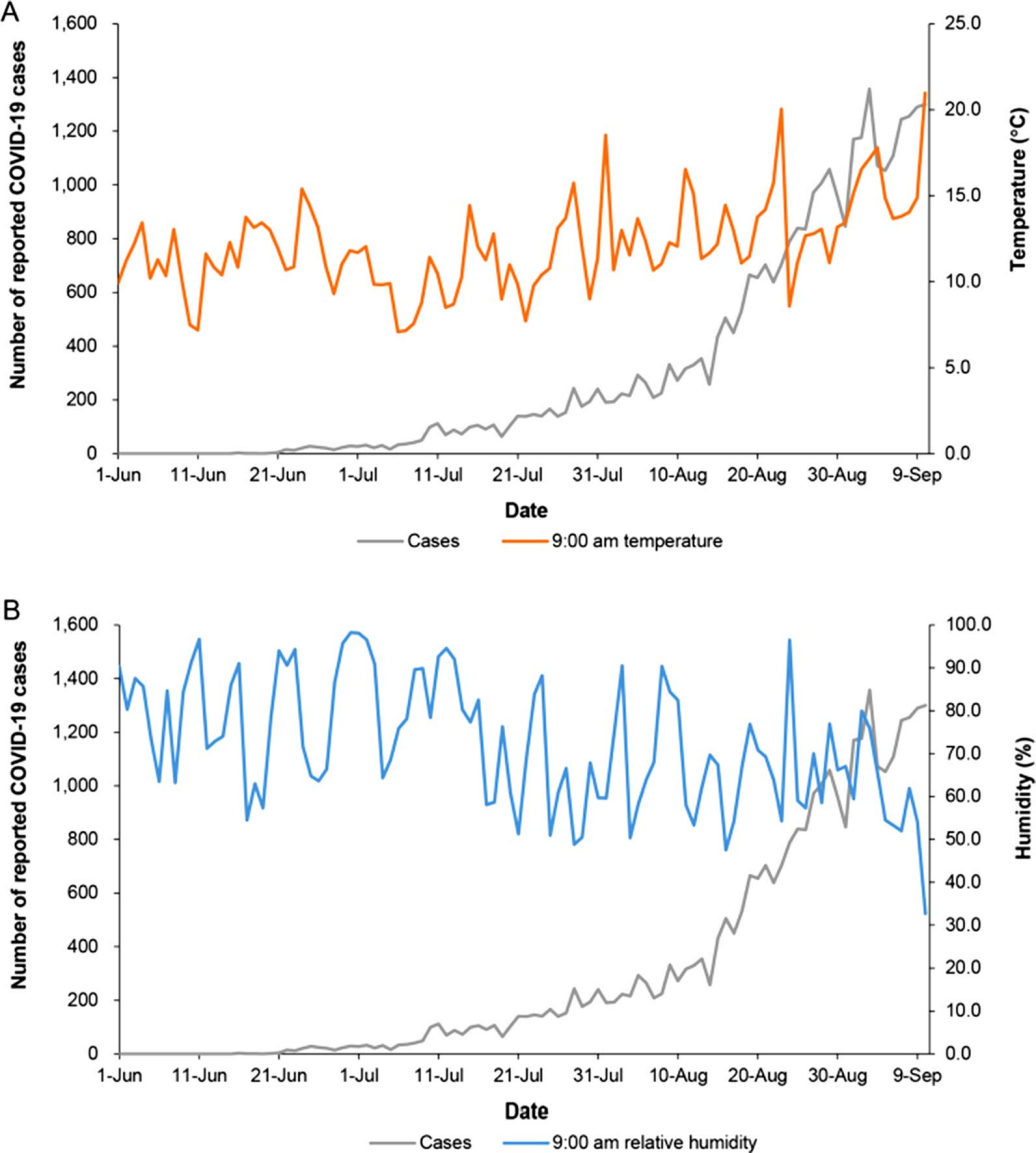
Reported cases of COVID-19 caused by the B.1.617.2 (delta) variant of SARS-CoV-2, Sydney, Australia 16 June to 10 September 2021 (primary axis) and 9:00 am temperature (°C) and 9:00 am relative humidity (%) (secondary axes)

Between 16 June and 10 September 2021, 31,662 locally-acquired cases with a residence within the five target LHDs were notified, excluding 6 and 191 cases in which infection was acquired interstate and overseas, respectively. During this period, relative humidity was variable, ranging from < 30% to 98%, and temperatures were mild (approximately 11–17 °C) (Table 1). The relationship between 9:00 am temperature and reported cases, and 9:00 am relative humidity and reported cases, as examples are shown in Fig. 2. In this outbreak during the period 16 June to 10 September there was a strong positive correlation between reported cases and the daily number of vaccine doses administered [Spearman rank correlation (ρ) = 0.81, *P* < 0.001] and daily number of COVID-19 tests performed (ρ = 0.95, *P* < 0.001). These metrics increased from approximately 30,000 to 120,000 doses per day, and 17,000 to 148,000 tests per day, during the period. The relationship between reported cases and the mobility index for Sydney was strongly negative (ρ = − 0.86, *P* < 0.001); the mobility index dropped from 0.46 to 0.09 during the study period. Thus, there was a strong response to the occurrence of this outbreak.

**Table 1:** Summary statistics of weather variables, Sydney, Australia from 16 June to 10 September, 2021, used in a study of an outbreak of COVID-19 caused by the B.1.617.2 (delta) variant of SARS-CoV-2

Variables	Minimum	2.5th percentile	Median	Mean	97.5th percentile	Maximum
9:00 am humidity, %	32.7	49.6	69.8	72.1	96.6	98.2
3:00 pm humidity, %	21.3	27.8	47.2	50.9	91.7	98.4
9:00 am temperature, °C	7.1	7.3	11.8	12.1	18.1	21.0
3:00 pm temperature, °C	9.1	11.6	17.1	17.2	23.0	26.3
Humidity difference, %	0.3	3.0	24.8	23.1	43.1	53.6
Temperature difference, °C	0.1	0.5	5.0	5.2	9.2	11.4

### Relationship between COVID-19 and weather

**Fig. 3:**
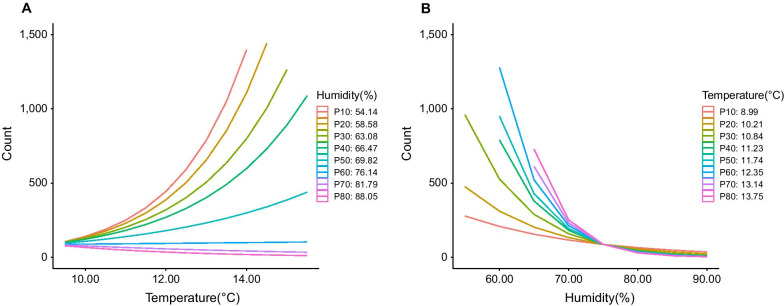
Interaction plots between 9:00 am temperature and 9:00 am relative humidity in a model of reported cases (counts) of COVID-19 caused by the B.1.617.2 (delta) variant of SARS-CoV-2, Sydney, Australia from 16 June to 10 September 2021. Interactions are shown using deciles of humidity (left, **A**) and temperature (right, **B**). The different lines represent the percentiles indicated

Based on correlation coefficients (Table 2), 9:00 am temperature, 9:00 am humidity and the difference between 9:00 am and 3:00 pm humidity were selected for further analysis; the latter did not show significance in univariable modelling (*P* = 0.621; Table 3). In multivariable modelling, four models with 9:00 am humidity, 9:00 am temperature or an interaction between these variables were fit to the data (Table 4). The model which included the interaction term showed the best fit (Table 5). Lower humidity was a consistent driver of COVID-19 cases caused by the SARS-CoV-2 delta variant, with a slight modification due to a significant interaction term (from − 0.110 to − 0.185, setting 9:00 am temperature at its median value). In contrast, the impact of higher temperatures on increased COVID-19 cases was mostly affected by the interaction term [from 0.25 (significant) to 0.04 (almost non-significant), setting 9:00 am humidity at its median value]. These exposure-response curves indicate that when humidity is high there is no effect of temperature on cases (Fig. 3A). However, when humidity is low, high 9:00 am temperatures are associated with more cases. But it should be noted that the range of 9:00 am temperature values during the study period (2.5th–97.5th percentile range: 7.3‒18.1 °C) was relatively narrow and temperatures were mild, favorable conditions for COVID-19 transmission. Relative humidity remained negatively associated with cases no matter whether the 9:00 am temperature was high or low. The effect of lower relative humidity on increased cases was more pronounced below relative humidity values of about 70% (Fig. 3B); below this threshold, not only are the effects of humidity pronounced but also the relationship between temperature and cases of the delta variant becomes apparent (Fig. 3A).

**Table 2:** Spearman correlation coefficient matrix among weather variables used to model reported cases of COVID-19 caused by the B.1.617.2 (delta) variant of SARS-CoV-2, Sydney, Australia from 16 June to 10 September, 2021

Variables	9:00 am temperature	9:00 am humidity	3:00 pm temperature	3:00 pm humidity	Temperature difference	Humidity difference
9:00 am temperature	1	− 0.53*	0.64*	− 0.33*	− 0.15	− 0.10
9:00 am humidity	‒	1	− 0.31*	0.63*	0.11	0.24*
3:00 pm temperature	‒	‒	1	− 0.57*	0.59*	0.42*
3:00 pm humidity	‒	‒	‒	1	− 0.47*	− 0.41*
Temperature difference	‒	‒	‒	‒	1	0.69*
Humidity difference	‒	‒	‒	‒	‒	1

*Significant bivariate correlation, *P* value < 0.05

**Table 3:** Univariable analysis of the association between weather factors and reported cases of COVID-19 caused by the B.1.617.2 (delta) variant of SARS-CoV-2, Sydney, Australia from 16 June to 10 September, 2021

Factor	Estimate (95% *CI*)	Likelihood ratio	*P*-value
9:00 am humidity	− 0.15 (− 0.18, − 0.12)	− 10.49	< 0.001
9:00 am temperature	0.68 (0.52, 0.85)	8.00	< 0.001
Humidity difference	− 0.03 (− 0.15, 0.09)	− 0.49	0.621

*CI* confidence interval

**Table 4:** Multivariable analysis of weather factors (14-day exponential moving average) and reported cases of COVID-19 caused by the B.1.617.2 (delta) variant of SARS-CoV-2, Sydney, Australia from 16 June to 10 September, 2021

Model	Factor	Estimate (95% *CI*)	Likelihood ratio	*P*-value
1	Intercept	16.37 (14.31, 18.44)	15.52	< 0.001
	9:00 am humidity	− 0.15 (− 0.18, − 0.12)	− 10.49	< 0.001
2	Intercept	− 2.74 (− 4.76, − 0.73)	− 2.66	0.008
	9:00 am temperature	0.68 (0.52, 0.85)	8.00	< 0.001
3	Intercept	10.24 (5.03, 15.45)	3.86	< 0.001
	9:00 am humidity	− 0.11 (− 0.15, − 0.07)	− 5.30	< 0.001
	9:00 am temperature	0.25 (0.03, 0.47)	2.21	0.027
4	Intercept	− 34.29 (− 51.80, − 16.78)	− 3.84	< 0.001
	9:00 am humidity	0.52 (0.27, 0.76)	4.20	< 0.001
	9:00 am temperature	4.23 (2.76, 5.70)	5.65	< 0.001
	9:00 am humidity × 9:00 am temperature	− 0.06 (− 0.08, − 0.04)	− 5.34	< 0.001

*CI* confidence interval

**Table 5:** Comparison of three multivariable models of the association between weather factors (14-day exponential moving average) and reported cases of COVID-19 caused by the B.1.617.2 (delta) variant of SARS-CoV-2, Sydney, Australia from 16 June to 10 September, 2021

Model	Parameter	AIC	Comparison	Likelihood ratio	*P*-value
1	Intercept	1,137.50	‒	‒	‒
	9:00 am humidity				
2	Intercept	1,151.62	Model 2 vs Model 3	18.53	< 0.001
	9:00 am temperature				
3	Intercept	1,135.09	Model 1 vs Model 3	4.40	0.036
	9:00 am humidity				
	9:00 am temperature				
4	Intercept	1,119.64	Model 3 vs Model 4	17.46	< 0.001
	9:00 am humidity				
	9:00 am temperature				
	9:00 am humidity × 9:00 am temperature				

*AIC* Akaike information criterion measure of model fit

The sensitivity analysis confirmed that the results of modelling were robust (Table 6).

**Table 6:** Sensitivity analysis for the comparison of three multivariable models of the association between weather factors (14-day exponential moving average) and reported cases of COVID-19 caused by the B.1.617.2 (delta) variant of SARS-CoV-2, Sydney, Australia from 16 June to 10 September, 2021

Model	Factor	Days used for exponential moving average								
		7-day			14-day			21-day		
		Estimate (SE)	Z	*P*-value	Estimate (SE)	Z	*P*-value	Estimate (SE)	Z	*P*-value
1	Intercept	14.59 (1.13)	12.89	< 0.001	16.37 (1.06)	15.52	< 0.001	17.30 (0.82)	21.16	< 0.001
	9:00 am humidity	− 0.13 (0.02)	− 8.23	< 0.001	− 0.15 (0.01)	− 10.49	< 0.001	− 0.16 (0.01)	− 14.55	< 0.001
2	Intercept	− 1.03 (0.05)	− 22.3	< 0.001	− 2.74 (1.03)	− 2.66	0.008	− 3.64 (0.86)	− 4.23	< 0.001
	9:00 am temperature	0.54 (0.003)	158.0	< 0.001	0.68 (0.09)	8.00	< 0.001	0.77 (0.07)	10.70	< 0.001
3	Intercept	3.89 (0.12)	33.18	< 0.001	10.24 (2.66)	3.86	< 0.001	11.28 (2.29)	4.93	< 0.001
	9:00 am humidity	− 0.05 (0.001)	− 44.97	< 0.001	− 0.11 (0.02)	− 5.30	< 0.001	− 0.12 (0.02)	− 7.00	< 0.001
	9:00 am temperature	0.40 (0.005)	86.44	< 0.001	0.25 (0.11)	2.21	0.027	0.25 (0.10)	2.55	0.011
4	Intercept	− 8.33 (8.05)	− 1.03	0.301	− 34.29 (8.93)	− 3.84	< 0.001	− 42.85 (7.65)	− 5.60	< 0.001
	9:00 am humidity	0.13 (0.11)	1.22	0.224	0.52 (0.12)	4.20	< 0.001	0.66 (0.11)	6.10	< 0.001
	9:00 am temperature	1.67 (0.66)	2.55	0.011	4.23 (0.75)	5.65	< 0.001	5.17 (0.66)	7.83	< 0.001
	9:00 am humidity × 9:00 am temperature	− 0.02 (0.01)	− 2.05	0.040	− 0.06 (0.01)	− 5.34	< 0.001	− 0.07 (0.01)	− 7.48	< 0.001

## Discussion

During the outbreak of COVID-19 in Sydney, beginning June 2021 and caused by the B.1.617.2 (delta) variant of SARS-CoV-2, low humidity was consistently associated with reported cases. Furthermore, and with implications for the response to outbreaks caused by the delta variant, we identified a threshold effect of low relative humidity (< 70%) whereby warmer, drier conditions might promote transmission of the SARS-CoV-2 delta variant. In addition to vaccination, stronger implementation of other interventions—such as mask-wearing and social distancing—might need to be considered during these risk periods to control outbreaks of COVID-19 caused by the delta variant.

Previously, in 2020, we identified a relationship between the original SARS-CoV-2 and relative humidity [8, 9]. In these earlier studies, a 1% decrease in relative humidity was predicted to increase cases in the range of 6–8%. In contrast, in the current study in the univariable model which included 9:00 am humidity, a 1% decrease in relative humidity was predicted to increase cases by 16.9% (setting 9:00 am temperature at its median value). Although outbreaks in Sydney in 2020 and 2021 cannot be directly compared, results suggest that the influence of humidity on transmission of the delta variant might be greater than that for the original SARS-CoV-2. The SARS-CoV-2 delta variant emerged recently and it has spread globally. Reports suggest that it might be more than twice as transmissible as the original SARS-CoV-2 that emerged in 2019–2020 [22]. To our knowledge there are no published studies specifically focusing on the relationship between transmission of the delta variant and weather. Further studies are needed to confirm the stronger association found in this study between transmission of the delta variant and humidity, and whether increased transmissibility might be partly explained by weather factors. This might provide useful information for policymakers to control transmission of the delta variant, for example by increasing indoors relative humidity to more than 70% in high-risk environments during times of the year in which transmission of this variant is favored.

The effect of humidity on transmission of SARS-CoV-2 virus has received substantial attention during 2020 and 2021, including studies conducted in Bangladesh [10] and China [4, 23, 24], and systematic reviews on the topic [25]. However, no consistent conclusion has been made. In several studies from 166 countries [24], China and the US [26] and Bangladesh [10], high relative humidity was found to be associated with a reduction in the daily number of COVID-19 cases or the effective reproductive number of SARS-CoV-2. In 30 Chinese provincial capitals, Liu et al. [23] found that low humidity likely favors SARS-CoV-2 transmission. In addition, using quantitative time-series analysis techniques, Qi et al. [4] estimated that for every 1% increase in relative humidity, daily confirmed cases decreased by 11% to 22% when temperature was in the range of 5.04‒8.2 °C. In the current study, we also found that humidity was negatively related to daily COVID-19 cases and was a stable driver of SARS-CoV-2 transmission, consistent with the above studies. However, the observed relationship between COVID-19 cases and humidity has not always been consistent; for example, this relationship was found to be heterogeneous between different cities in China [12], and in a global study of 190 countries an inverse J-shaped relationship was found between relative humidity and COVID-19 incidence, in which risk was greatest at 72% relative humidity [7]. It is likely that a range of other factors influences the relationship between transmission of SARS-CoV-2 and humidity, particularly climatic zone. Also, the various control strategies implemented—such as mandatory mask-wearing, social distancing, testing and vaccination—make characterization of the relationship between weather factors and SARS-CoV-2 transmission within outbreak situations challenging.

The relationship between temperature and COVID-19 cases has not yet been fully characterized. In a study in China, no relationship between temperature and COVID-19 cases was found [27]. A negative correlation between temperature and COVID-19 cases—less transmission at higher temperatures—has been reported by Xie et al. [28] and Notari et al. [29]. However, in our study, a positive correlation between temperature and COVID-19 cases caused by the SARS-CoV-2 delta variant was found, but only in the situation when relative humidity values are around 70% or lower. This is consistent with positive associations between average temperature and daily COVID-19 cases reported in nine Asian cities [30].

The interactions among weather factors might provide a reasonable explanation for the above contradictory results. Previous research has suggested a potential interaction between relative humidity and temperature and COVID-19 case reports, but the exact mechanism of the interaction is unclear [4]. This might be due to both temperature and humidity affecting the function of the respiratory mucosa as a barrier to the virus and infection, and hence affecting the spread of SARS-CoV-2 [31]. The same phenomenon has been previously described in influenza studies: temperature was inversely associated with influenza and the relationship could be modified by humidity [32]. Hence the suggestion has been made that COVID-19 might develop into a seasonal disease [33]. However, studies on the correlation between weather factors such as temperature and humidity and their interaction and transmission of the SARS-CoV-2 delta variant have not yet been reported, and further research is urgently needed to support policy and control. Our results suggest that weather could be a more important consideration during outbreaks of this delta variant. Therefore, it is important in future research to focus on those specific periods in which transmission might be increased to better understand the mechanisms involved and how public health advice and interventions might be targeted. Given the advances that have been made in forecasting seasonal influenza outbreaks [34], the same approach can be anticipated for seasonal COVID-19 once the mechanisms of spread are better understood.

In this study we assumed that cases were infected within their LHD and that temperature and humidity measured at meteorological recording stations was a proxy for the conditions experienced when transmission occurred. More precise measures of exposure are difficult to access in the field and within an outbreak setting. Temperature and humidity measurements represent outdoors conditions, and so are a proxy for the conditions experienced by the population exposed to infection. We also assumed that case reporting in this outbreak was high and that differential bias was not present. During this outbreak high levels of testing occurred—an average daily testing rate of > 80,000 tests were reported—and confirmation rates remained consistent [16], so reporting bias is likely small. Although observational studies such as the current one suffer from measurement and information biases, the coherence of evidence from a growing number of studies strengthens the hypothesis that SARS-CoV-2 transmission is influenced by weather.

## Conclusions

To date, countries in the northern hemisphere have not experienced large outbreaks of COVID-19 caused by the delta variant of SARS-CoV-2 during the winter months. The present study contributes to our growing knowledge of the relationship between SARS-CoV-2 transmission and weather, and specifically the transmission of the delta variant, which is currently the dominant variant globally. It suggests that both relative humidity and temperature play a role via an interactive effect. In similar climatic zones to this study in which low humidity and mild temperatures are experienced during the winter months, the control of the delta variant might be more difficult than previously expected. Increasing the ambient humidity (e.g., > 70%) might be a useful alternative measurement for reducing the transmission risk of the delta variant of SARS-CoV-2 in the future, and during periods of anticipated increased risk (such as the winter months) additional disease control interventions might be warranted.

## Supplementary Information

**Additional file 1.** Data used to analyse the association between weather variables and reported cases of COVID-19 caused by the B.1.617.2 (delta) variant of SARS-CoV-2 in Sydney, Australia between June and September 2021.

## Data Availability

All data analysed during this study are included in this published article (and its Additional files).

## Abbreviations

AIC: Akaike information criterion
EMA: Exponential moving average
GAM: Generalized additive model
LHD: Local health district
ρ: Spearman rank correlation
